# Porous Carriers for Controlled/Modulated Drug Delivery

**DOI:** 10.4103/0250-474X.59540

**Published:** 2009

**Authors:** G. Ahuja, K. Pathak

**Affiliations:** Rajiv Academy for Pharmacy, NH #2, P. O. Chhattikara, Mathura-281 001, India

**Keywords:** Adsorbents, adsorption, controlled delivery, porosity, porous carriers

## Abstract

Considerable research efforts have been directed in recent years towards the development of porous carriers as controlled drug delivery matrices because of possessing several features such as stable uniform porous structure, high surface area, tunable pore size and well-defined surface properties. Owing to wide range of useful properties porous carriers have been used in pharmaceuticals for many purposes including development of floating drug delivery systems, sustained drug delivery systems. Various types of pores like open, closed, transport and blind pores in the porous solid allow them to adsorb drugs and release them in a more reproducible and predictable manner. Pharmaceutically exploited porous adsorbents includes, silica (mesoporous), ethylene vinyl acetate (macroporous), polypropylene foam powder (microporous), titanium dioxide (nanoporous). When porous polymeric drug delivery system is placed in contact with appropriate dissolution medium, release of drug to medium must be preceded by the drug dissolution in the water filled pores or from surface and by diffusion through the water filled channels. The porous carriers are used to improve the oral bioavailability of poorly water soluble drugs, to increase the dissolution of relatively insoluble powders and conversion of crystalline state to amorphous state.

The goal of any drug delivery system is to provide the therapeutic amounts of drug to the proper site in the body to achieve promptly and to maintain the desired drug concentration[1]. Delivery of drugs by means of controlled release technology began in the 1970s and has continued to expand rapidly[2]. Various drug delivery systems, such as liposomes, micelles, emulsions, polymeric micro/nanoparticles have been showing great promise in controlled and targeted drug delivery. Among these systems porous materials are emerging as a new category of host/guest systems[3–6].

Greater attention has been focused on the development of porous materials as controlled drug delivery matrices because of possessing several alternatives features such as stable uniform porous structure, high surface area, tunable pore sizes with narrow distribution and well defined surface properties[7,8]. Owing to wide range of useful properties, porous carriers have been used in pharmaceuticals for many purposes including development of novel drug delivery systems such as floating drug delivery system, sustained drug delivery system and improvement of solubility of poorly soluble drugs[9–14]. These materials possess vast amounts of nanopores that allow the inclusion of drugs[1]. These features allow them to adsorb drugs and release them in a more reproducible and predictable manner. The use of mesoporous, microporous and nanoporous carriers used for drug delivery is a part of growing research[15,16].

Liquid penetration into and its subsequent flow through such porous materials depends on both the molecular and the bulk property of the liquid and the geometric and surface property of the porous medium. The required displacement depends upon the pore size, surface tension of liquid and the contact between surface and liquid[17].

When a porous hydrophobic polymeric drug delivery system is placed in contact with the appropriate dissolution medium, release of drug to medium must be preceded by the drug dissolution in the water filled pores or from surface and by diffusion through the water filled channels[18]. Drug release from the porous carrier may be complete within 10 min or be incomplete after several hours or days. Solvent polarity and surface properties play an important role in the adsorption and release from the porous carriers[19,20].

Adsorption is the interphase accumulation of concentration of substances at a surface or interface. Adsorption is the phenomenon by which the molecules of gas, vapour and liquid spontaneously concentrate at a contacting surface without undergoing chemical reaction, thereby forming a surface or interfacial layer. Molecules that adsorbed on the solid surfaces are referred as adsorbate and the surface to which they are adsorbed referred as substrate and adsorbent.

## Advantages of adsorption for drug delivery:

Adsorption and entrapment of drug molecules in carrier granules leads to an enhancement of physicochemical stability of the drug. The presence of larger number of hydroxyl groups that form inter- and intra-molecular hydrogen bonded structure were identified as a factor for enhancing dissolution. Adsorption is readily adoptable for thermolabile drugs and carriers. Improved dissolution rates are attributed to decreased drug particle size with a consequent increase in the surface area and to increase in the thermodynamic activity of drug in dispersed state.

## Porosity:

**Fig. 1:**
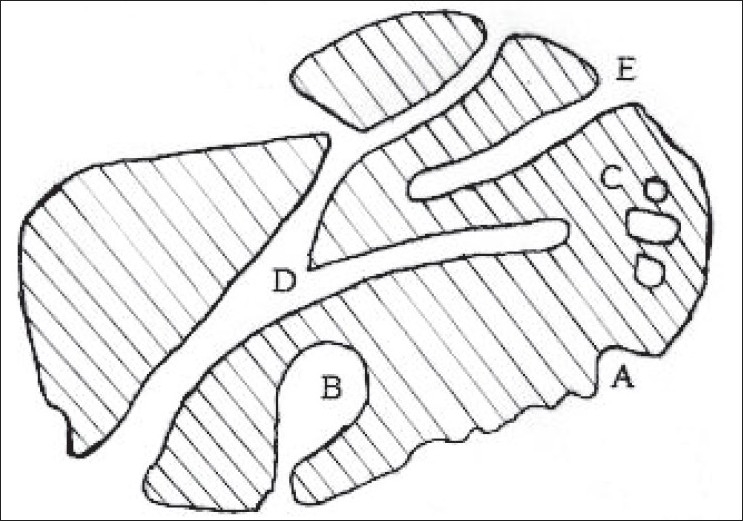
Magnified physical picture of a porous solid particle Physical picture of porous solid showing A- surface roughness, Bink bottle (blind pores), C- closed pores, D- transport pores (pores through the solid) and E- cylindrical blind

The word pore comes from the Greek word ‘*πoρoσ*’, which means passage. This indicates the role of a pore acting as a passage between the external and the internal surfaces of a solid, allowing material, to pass into, through or out of the solid. Fig. 1 represents the physical picture of porous solid and various types of pores that may occur in a solid. It is probable that pores are irregular in shape and may also be interconnected. An open pore is one which is connected to the external surface of a solid and allows the passage of an adsorbate through the solid in contrast to the closed pore that is a void within the solid which is not connected to the external surface and hence is isolated. Third type of pore is the transport pore that connect different parts of the external surface of the solid to the inner micro porosity and finally the blind pores that are connected to the transport pores but do not lead to any other pore or surface. Porosity is the collective term for these pores and their distribution in the structure of the solid. Based on the pore size the porosity is classified as microporosity, mesoporosity and macroporosity as given in Table 1.

**TABLE 1:** CLASSIFICATION OF POROUS MATERIALS/CARRIERS FOR DRUG DELIVERY[21]

Types of Pores	Pore Dimensions	Pore Formation
Microporous	Width less than 2 nm	Formed as a result of imperfect stacking of constituent molecules
Mesoporous	Width between 2 and 50 nm	Result of major defects in the structure
Macroporous	Width greater than 50 nm	Formed as a result of Major lattice structure defects such as racks, fissures and etching channels.

## Microporosity:

The micropores are formed as the result of imperfect stacking of constituent molecules and packing arrangements of the bulk material, producing a lack of crystallite alignment, and small pseudo-graphitic crystallites. Their shape has been shown, by TEM studies to be either slit-like or convoluted in shape. Adsorption into micropores is completely reversible. The class of micropores may be subdivided into three separate groups; ultramicropores, micropores and supermicropores. Ultramicroporosity (diameter < 0.5 nm) is usually responsible for activated diffusion and the pore diameter is comparable to that of the adsorbate molecule. Microporosity (diameter~0.5-1.4 nm) fills quickly, within the first few minutes of adsorption and overlap of the pore wall potentials is the pore filling mechanism for adsorption in micropores. Supermicroporosity (size-1.4-2.0 nm) promotes co-operative pore filling wherein monolayer formation occurs and the pore diameter is effectively reduced enhancing the adsorption potential of the pore hence increasing adsorption and completing the pore filling at low relative pressure.

The micropores provide sites of maximum adsorption potential for an adsorbed molecule/atom and within the pore. Due to the close proximity of the walls of micropores an interaction of the polyionic potentials, the result of overlapping of the dispersion fields may occur resulting in a relatively deep potential energy well and enhanced adsorption at a given pressure. Hence, diffusion into the ultramicropores has a significant activation energy associated with it. The pore filling process may be divided into three steps, first is the monolayer formation, second the pore filling by co-operative effects and third is completion of the pore filling process.

## Mesoporosity:

Mesopores are the result of major defects in the structure of a solid and serve as passages, providing a transport system, to the micropores. These are the pores which give rise to the phenomenon of capillary condensation. The mesopores fill by multilayer formation. The pore diameters, greater than 2 nm but less than 50 nm according to IUPAC definition, are so large that at low relative pressures monolayer coverage occurs followed by further layers and the adsorbed film acts as a nucleus upon which capillary condensation may take place.

## Macroporosity:

The micropores are considered important in the process of adsorption whereas the meso- and macropores primarily act as transport pores. Major lattice structure defects, such as racks, fissures and etching channels, within a solid lead to the formation of macropores which may be treated as an open surface. It is possible to observe macroporosity by optical microscope and scanning electron microscopy as they are of the order 50 nm and greater. There is no actual upper limit to the diameter of the pores but it is usually 1-2 mm[21].

Pharmaceutically exploited porous adsorbents include ethylene vinyl acetate (macroporous) alumina, silica (mesoporous), clay and zeolites, activated carbon, porous silicon dioxide, propylene foam powder, porous calcium silicate (microporous), magnesium aluminometa silicate, porous ceramics, calcium carbonate, iron oxides, bauxite, zirconium oxide, titanium dioxide (nanoporous)[22] and other mixed oxides. Structures of various adsorbents are available at the Max-Plank Institute website[23].

## METHODS OF DRUG LOADING

Coating the drug-loaded core particles with a polymer film is one of the most widely investigated technologies to make multi-particulate dosage forms. A large variety of materials like Nonpareil® sugar beads and Celphere® crystalline cellulose, have been applied as core particles for this technology. However, the drug-loading ratio is relatively low, since the loading area is limited to the outer surface of core particles. To overcome these pharmaceutical difficulties, there has been increasing interest in the use of porous materials as the drug-loading core by making use of their high surface area[24]. Drug-loaded particles appear particularly suited for controlled release and drug targeting. These systems are expected to enhance the bioavailability of poorly absorbed drugs, entailing a lowering of the therapeutic dose.

### Simple mixing:

In this method, adsorbent is placed in the drug solution and stirred for suitable time using magnetic stirrer. The solution is then allowed to stand for 1 h, separated and dried over 24 h at 60°. This method is used for the variety of drugs like ibuprofen, dexamethasone, griseofulvin, ranitidine and furosemide[14].

### Solvent evaporation:

Adsorbent is closely sieved in the range of 250-350 μm to nullify the effect due to variation in particle size. Drug was loaded in solvent followed by the constant addition of the adsorbent, kept to evaporate under ambient conditions[7].

### Loading under high pressure:

**Fig. 2:**
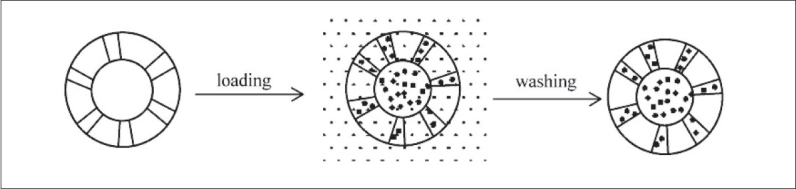
Diagrammatic representation of the drug loading in porous adsorbent under high pressure[1] Drug loading under high pressure: (i) adsorbent (air is adsorbed in the adsorbent); (ii) drug gets loaded into the inner core of the adsorbent under high pressure; (iii) the drug loaded adsorbent.

Drug were mixed with the adsorbent in sufficient ratio and put into the high pressure adsorption equipment (fig. 2) for a period over 24 h. After being washed with a deionised water to get a rid of unentrapped drug, the powder was dried in vaccum oven at 65° for 5 h. This method is used for the loading of Brilliant Blue[25].

### Vaccum process:

Adsorbent is placed in the drug solution and the mixer evacuated for suitable time after which the vaccum was released. The adsorbent and drug solution were then allowed to stand for 1 h. Following this, solids separated using filter paper and dried for 24 h at 60°. Various drugs like diltiazem hydrochloride, benzoic acid, sodium benzoate are used for loading on the adsorbent[14]. In another method, drug and adsorbent are mixed in the suitable volatile solvent for 6 h and evaporated the obtained mixer under reduced pressure. The obtained powder was dried in vaccum for 3 h. This method is used for the loading of hydrophobic drugs like phytonadione[26].

### Stirring in drug solution or suspension:

Adsorbent is stirred in drug solution or suspension followed by drying in simple tray drier. Instead of using excess medium requisite minimum drug solution or suspension were loaded. Therefore vaccum process is not essential and high yield can be achieved. This method can be applied to a variety of drugs like theophylline[24].

### Layer- by- layer adsorption (LbL):

The technique was usually performed in the aqueous solution at room temperature and thus it was suitable to encapsulate polypeptide and proteins drugs of poor stability. A LbL assembly of two oppositely charged polyelectrolytes at solid surfaces was developed as the alternating adsorption of these polyelectrolytes on a charged substrate due to their electrostatic attraction and the complex formation resulting in the defined macromolecular layer on the surfaces[1].

## PHARMACEUTICAL APPLICATIONS

### Improvement in dissolution:

Mesoporous materials offer a potential means to increase the dissolution of poorly soluble drug via effects on surface area or crystallinity. At pore sizes only a few times larger than the drug molecule, the formation of crystalline material is restricted by the confined space of the pores, thus retaining the drug in its noncrystalline, amorphous form. The amorphous form is known to exhibit higher dissolution rates than the crystalline phase[27].

### Chemical and pharmaceutical purification:

Adsorption in carbonaceous adsorbents is suitable for the decolourisation and purification of a wide range of organic and inorganic compounds including: amines, hydrochloric and other mineral acids, amino acids, glycols, hydrocarbons. Catalysts can be poisoned or fouled by low concentration organic compounds, sulfur or mercury species. Carbonaceous materials, non impregnated and impregnated types, are typically used as “guard beds” to protect the catalysts (from fouling and/or deactivation) and the equipment (from corrosion) in streams such as natural gas, acetylene, ethanepropane and ethylene oxide.

### Food purification and decolorization:

Adsorption in carbonaceous adsorbents is widely used for the decolourisation of natural and synthetic sweeteners, decolorization of cane sugar syrup, decolorization of vitamines and purification of glycerin.

### Improvement in bioavailability:

Low density porous carriers such as porous silicon dioxide (Sylysia), polypropylene foam powder (Accurel), porous calcium silicate (Florite), magnesium aluminometa silicate (Neusilin), and porous ceramic with open or closed pore structure that provide large surface area are used for the improvement of dissolution and bioavailability of poorly soluble drugs like meloxicam, aspirin, indomethcin[28].

### Solubility improvement:

Florite RE (FLR) is a porous calcium silicate that possesses many interparticle and intraparticle pores, particularly of sizes 12 and 0.15 μm, respectively, on its surface. FLR is easily dispersible in all aqueous fluids and has been used to adsorb oily and other drugs, as a compressive agent in pharmaceuticals, and to improve solubilitys[9].

### Improvement of surface affinity:

Otsuka *et al.*[26] investigated the surface-modification of silica gel with the silane coupling to improve the surface affinity to an oily medicine, phytonadione. However, a rapid release during the whole process, especially initial burst release has been observed in some cases when inorganic porous particles were used as the drug host.

### Sustained/controlled release:

Several porous minerals have been used including synthetic zeolite, silica xerogel materials, porous hollow silica nanoparticle, porous hydroxyapatite, porous silica–calcium phosphate composite, porous calcium carbonate microparticle and other porous ceramics. These materials possess vast amounts of nanopores that allow the inclusion of drugs in them and useful in making sustained/controlled release formulations[29–34]. Accurel MP 1000 is characterized by open porous network with pore size predominantly in the micro and mesoporous range. This microporous adsorbent has been evaluated for the development of floating drug delivery system and stem development[35]. A single unit floating drug delivery system consisting of low density microparticles were developed using porous carrier material (foam powder), drug and polymer, to increase the gastric residence time of drug delivery systems[36–39]. The floating behaviour of the low density drug delivery system could successfully be combined with accurate control of drug release.

Ohta *et al.*[24] investigated the use of Silica gel as core particles to design a simple preparation for controlled delivery system with high drug content. Drug loading was carried out by immersing the silica gel in a pre-heated drug solution or suspension. The drug-loaded silica gel was coated with hydroxypropylmethylcellulose (HPMC) and an aqueous dispersion of ethylcellulose (Aquacoat^®^) to control the drug release. Layer by Layer (LbL) self-assembly technique has been a powerful tool where polyelectrolyte multilayer films were elaborated on various particles through alternating deposition of oppositely charged polyelectrolytes mainly due to the electrostatic attraction. LbL adsorption on the porous carriers showed potential applications in controlled release, cosmetic for the delivery of the proteins like bovine serum albumin, glucose oxidase, urease and superoxide dismutase), as the depositing species to prepare bioactive core-shell particles. Ito *et al.*[28] developed the self emulsifying drug delivery system by using three kinds of adsorbents microporous calcium silicate (Florite™ RE), magnesium aluminometa silicate (Neusilin™ US_2_) and silicon dioxide (Sylysia™ 320) with low molecular weight heparin (LMWH). The results suggest that adsorbent system is useful as an oral solid delivery system of poorly absorbable drugs such as LMWH. Table 2 compiles the work done on drug release modulation by use of porous carriers.

**TABLE 2:** A COMPILATION OF THE WORK DONE ON DRUG RELEASE MODULATION BY USE OF POROUS CARRIERS OF PHARMACEUTICAL SIGNIFICANCE

Drug	Adsorbent(s)	Technique	Remark
Alendronate[41]	ydroxyapatite (HA)	Simple Mixing	Alendronate modified nanoparticles had a strong and specific adsorption to HA. The amount of nanoparticle adsorbed on to HA tend to be smaller when the content of alendronate was decreased and the large block length of monomethoxy PEG was found to reduce the potency of alendronate.
Ibuprofen[42]	MgO modified SBA-15 and pure Silica SBA- 15	Simple Mixing	The *in vitro* release study shows that surface modification greatly decreased the Ibuprofen release rate i.e. 63% in 6 h and the release was complete in 1 h from pure silica SBA-15. The surface modified with MgO created affinity with acidic Ibuprofen molecules and retarded the release rate from the mesoporous matrix.
Ibuprofen[7]	Microporous Polypropylene (Accurel MP 1000)	Solvent Evaporation	The drug release was investigated in phosphate buffer pH 7.2. All batches shows excellent *in vitro* floating property. Effect of solvent properties shows a positive influence on drug adsorption and release. Release profile of some batches could be considered as gastro retentive drug delivery system.
Low molecular weight Heparin[43]	Microporous Calcium Silicate (FloriteTM RE), Magnesium Aluminometasi-licate (NeusilinTM US2) and Silicon Dioxide (Sylysia TM 320).	Simple mixing	New oral solid dosage form of low molecular weight heparin has been prepared by using surfactant and adsorbents. This study has been done to improve the intestinal absorption of low molecular weight Heparin. Results shows that adsorbent system is used as an oral solid delivery system of poorly absorbable drugs such as low molecular weight Heparin.
Loratadine[44]	Porous Polystyrene Beads (PPB)	Solvent Evaporation	Porous Polystyrene Beads are potential carrier for solidification of SES, with high SES to PPB ratios required to obtain solid form. Geometrical features such as bead size and pore architecture of PPB was found to govern the loading efficiency and *in vitro* release from SES loaded PPB.
Insulin[45]	Poly (α, β-L Malic acid) (PMA) and water soluble Chitosan	Layer- by- Layer adsorption	This method can offer high drug loading capacity and high encapsulation efficiency. Also the release behaviour can be controlled since the cell thickness and its permeability, was readily adjusted on the nanometer scale by polyelectrolyte adsorption cycle. The Insulin loaded nanoparticles appears to be especially promising for the parenteral administration on insulin in diabetic patients.
Gentamycin[46] Sulphate	Microporous Bioactive Glass (MBG)	Immersion	The amount of drug loading was greatly influenced by the well ordered mesoporous structure and the amount of drug loading of MBG was three times more than that of conventional 58S. So the well ordered mesoporous bioactive glasses might be used as a bioactive drug release system for preparation of bone materials.
Ibuprofen[1]	Porous Calcium Carbonate	Vacuum Loading	In this work, polymer/inorganic hybrid core-shell microcapsules were fabricated for controlled release of poorly water-soluble drugs. The porous inorganic particles are useful to load drugs in amorphous state and the polyelectrolyte multilayer films coated on the particle assuage the initial burst release.
Dexamethasone[22]	Nanoporous Titanium Dioxide (TiO_2_)	Immersion	This novel drug-loading scheme on a biocompatible surface, will benefit patients who require the deployment of drug-eluting implants. Anticoagulants, analgesics and antibiotics for drug delivery during the time of maximal pain or risk for patients undergoing orthopaedic procedures.
Theophylline[24]	Silica Gel	Immersion in drug solution and suspension	Evaluation of the drug-loading process indicates that drug deposition in the pores occurs during the loading process and the drug-loading efficacy is strongly related to the drug solubility. An HPMC undercoating effectively suppresses the drug release, as it smoothes the drug-loaded core surface and aids in the formation of a continuous Aquacoat® coating film.
Antipyrine, Ibuprofen, Griseofulvin, Ranitidine and Furosemide[27]	Mesoporous Silicon	Simple Mixing	When the dissolution rate of the free/unloaded drug was high, the microparticles caused a delayed release. However, with poorly dissolving drugs, the loading into the mesoporous microparticles clearly improved dissolution. In addition, pH dependency of the dissolution was reduced when the drug substance was loaded into the microparticles.
Ibuprofen[20]	Mesoporous Silica (MCM 41)	Simple Mixing and Solvent Evaporation	For their surface areas and ordered mesoporous structure, micelle templated silica materials constitute a potentially interesting drug carrier for non- water soluble drug carrier. The association of nano-structured mineral to the molecular state of the drug presents a great interest for pharmaceutical application as it allows a control of the kinetic delivery of the lipophillic drugs.
Fenofibrate[47]	Poly (ethylene glycol)- block- poly (€- caprolactone)	Direct Dissolution, Solvent Evaporation and Dialysis	PEG-b-PCL in acetonitrile assembles with fenofibrate in to drug loaded polymeric micelles with the addition of water and the subsequent removal of negative ACN-Water azeotrope.
Haemoglobin[48]	Mesoporous Ceramics	Adsorption	This study demonstrates that the haemoglobin adsorbed aquasomes can carry the oxygen satisfactorily and it also establishes the superiority of haemoglobin aquasomal formulation as artificial blood substitute.
Brilliant Blue (BB)[2]	Porous Hollow Silica	Loading under high pressure	After being loaded into the inner core and on the surfaces of the nanoparticles, BB was released slowly into a bulk solution for about 1140 min as compared to only 10 min for the normal SiO_2_ nanoparticles, thus exhibited a typical sustained release pattern without any burst effect. It showed that PHSN have a promising future in controlled drug delivery applications.
BAY 129566, Naproxen, Ketoprofen, Indomethacin, Testosterone[49]	Magnesium aluminometasilicate (Neusilin US2)	Hot melt Granulation	A competitive balance between hydrogen bonding of the drugs with Neusilin and Ostwald ripening determines drug dissolution from solid- dispersion granules upon storage.
Verapamil Hydrochloride[12]	Polypropylene	Solvent Evaporation	The size of microparticles was almost independent of the amount of the drug loading, but strongly depends on the amount of polymer. The drug was partly dissolved and partly in the amorphous form distributed throughout the system.
Benzoic Acid, sodium Benzoate, Diltiazem[14]	Porous Ceramics- N-light N3, Starlight SLK 1000 and Carbolite 16/20	Vacuum Loading	The drug loading was influenced by the solution concentration and by the porosity and bulk density of ceramic. *In vitro* dissolution testing of the loaded porous microparticles showed an initial burst release of each drug followed by sustained releases. The release was influenced by the surface.
Sodium Ampicillin[50]	Hydroxyapatite (HA) and Glass reinforced hydroxyapatite (GR- HA)	-	The release kinetics studies in which where a large amount of sodium ampicillin was released, followed by slower release rate and then a final stage where the release amount approaches zero have indicated that the possibility of using these materials as possible carriers for drug delivery.
Dexamethasone[51]	Poly (dl, lactide-co- glycolide) (PLGA)	Simple Mixing	The gelatine coated PLGA microspheres has higher interaction with fibronectin compared with the other gelatine surface modified PLGA microspheres. Dexamethasone was released slowly from gelatine surface modified PLGA microspheres.
Phytonadione (VK1)[26]	Silica Gel	Solvent Evaporation and Vacuum loading	The VK1 release from the modified silica gels was initially rapid, slowed markedly after 1 h, and continued for more than 24 h. The amount of VK1 released from the modified surface silica gels by C7, C18 or F3 increased with increasing density of the surface modification group. The mean drug release moment (MDT) decreased with an increase in surface-modified group density.
Vasopressin[52]	Accurel® (polypropylene)	-	The present module is a novelty among the known membrane controlled drug delivery reservoir systems, since it has been loaded by an unsaturated drug solution and because the encapsulating membrane is not the sole release rate controlling factor.
Potassium Chloride[18]	Porous Ethyl Cellulose	Solvent Evaporation	Release of water soluble drug from polymeric system of known void fraction may be explained by the considering a dissolution- controlled regime.

### Others applications:

Porous ceramics are bodies comprised of a three-dimensional array of hollow polygons, known as cells. If the individual cells are interconnected the porous ceramic is termed open-cell, while if they are isolated from each other, the porous ceramic is termed closed-cell. They can also be partly open or partly closed. These properties have found many applications including catalyst supports, filters for molten metals and hot gases, refractory linings for furnaces, and porous implants in the area of biomaterials[40]. Crystalline microporous materials generally have a narrow pore size distribution. This makes it possible for a microporous material to selectively allow some molecules to enter its pores and reject some other molecules that are either too large or have a shape that does not match with the shape of the pore. A number of applications involving microporous materials utilize such size and shape selectivity.

## CONCLUSIONS

Today the porous carriers have a major role to play in the pharmaceutical industry. Their inner structure consists of unidirectional channel like pores that forms a hexagonal pattern. The presence of porous structure like microporous, mesoporous and macroporous structure was found to be essential in providing the sustained drug delivery systems, floating drug delivery systems and improvement of poorly water soluble drugs etc. Therefore, in the years to come, there is going to be continued interest in the porous carriers to have better materials for drug delivery systems.

## References

[1] Wang C, He C, Tong Z, Liu X, Ren B, Zeng F (2006). Combination of adsorption by porous CaCO 3 microparticles and encapsulation by polyelectrolyte multilayer films for sustained drug delivery. Int J Pharm.

[2] Li Z, Wen L, Shao L, Chen J (2004). Fabrication of porous silica nanoparticles and their applications in release control. J Control Release.

[3] Benita S (2004). Microencapsulation, Drugs and the Pharmaceutical Science.

[4] Brigger I, Dubernet C, Couvreur P (2002). Nanoparticles in cancer therapy and diagnosis. Adv Drug Deliv Rev.

[5] Torchilin VP (2005). Recent advances with liposomes as pharmaceutical carriers. Nat Rev Drug Discov.

[6] Allen TM, Cullis PR (2004). Drug delivery systems: Entering the main stream. Science.

[7] Sher P, Insavle G, Porathnam S, Pawar AP (2007). Low Density porous carrier drug adsorption and release study by response surface methodology using different solvents. Int J Pharm.

[8] Shivanand P, Sprockel OL (1998). A controlled porosity drug delivery system. Int J Pharm.

[9] Sharma S, Sher P, Badve S, Atmaram PP (2005). Adsorption of Meloxicam on porous calcium silicate: Characterisation and tablet formulation. AAPS Pharm Sci Tech.

[10] Streubel A, Sipemann J, Bodmeier R (2003). Floating matrix tablets based on low density foam powder: Effects of formulation and processing parameters on drug release. Eur J Pharm Sci.

[11] Yuasa H, Takashima Y, Kanaya Y (1996). Studies on the development of intragastric floating and sustained release preparation: I: Application of calcium silicate as a floating carrier. Chem Pharm Bull (Tokyo).

[12] Strebuel A, Siepmann J, Bodmeier R (2002). Floating microparticles based on low density foam powder. Int J Pharm.

[13] Salis A, Sanjust E, Solinas V, Monduzzi M (2003). Characterization of Accurel MP 1004 polypropylene powder and its use as a support for lipase immobilization. J Mol Cat B Enz.

[14] Byrne RS, Deasy PB (2002). Use of commercial porous ceramic particles for sustained drug delivery. Int J Pharm.

[15] Song SW, Hidajat K, Kawi S (2005). Functionalized SAB-15 material as carrier from controlled drug delivery: Influence of surface properties on matrix drug interactions. Langmuir.

[16] Andersson J, Rosenhoim J, Areva S, Linden M (2004). Influences of material characteristics on ibuprofen drug loading and release profiles from ordered micro- and mesoporous silica matrices. Chem Mater.

[17] Faruk C (2001). Scale effect on porosity and permeability: Kinetics model and correlation. Am Inst Chem Engg J.

[18] Gurny R, Doelker E, Peppas NA (1981). Modelling of sustained release of water soluble drugs from porous, hydrophobic polymers. Biomaterials.

[19] Gren T, Bjerre C, Camber O, Ragnarsson G (1996). *In vitro* drug release from porous cellulose matrices. Int J Pharm.

[20] Charney C, Begu S, Tourne P, Nicole L, Lerner D, Devoisselle JM (2003). Inclusion of ibuprofen in mesoporous template silica: Drug loading and release property. Eur J Pharm Biopharm.

[21] Fletcher AJ Porosity and sorption behaviour.

[22] Ayon AA, Cantu M, Chava K, Agrawal CM, Feldman MD, Johnson D (2006). Drug loading of Nanoporous TiO_2_ films. Biomed Mat.

[23] http://www.mpi-halle.mpg.de/~porous_m.

[24] Ohta KM, Fuji M, Takeri T, Chikazawa M (2005). Development of a simple method for the preparation of silica gel based controlled drug delivery system with high drug content. Eur J Pharm Sci.

[25] Li Z, Wen L, Shao L, Chen J (2004). Fabrication of porous silica nanoparticles and their applications in release control. J Control Release.

[26] Otsuka M, Tokumitsu K, Matsuda Y (2000). Solid dosage form preparations from oily medicines and their drug release: Effect of degree of surface modification of silica gel on the drug release from phytonadione-loaded silica gel. J Control Release.

[27] Salonen J, Laitinen L, Kaukonen AM, Tuura J, Bjorkqvist M, Heikkila T (2005). Mesoporous silicon microparticles for oral drug delivery: Loading and release of five model drugs. J Control Release.

[28] Ito Y, Kusawake T, Ishida M, Tawa R, Shibata N, Takada K (2005). Oral solid gentamicin preparation using emulsifier and adsorbent. J Control Release.

[29] Fisher KA, Huddersman KD, Taylor MJ (2003). Comparison of micro and mesoporous inorganic materials in the uptake and release of the drug model fluorescein and its analogues. Chem Eur J.

[30] Suzuki K, Yumura T, Tanaka Y, Akashi M (2001). Thermo-responsive release from interpenetrating porous silica-poly (N-isopropylacrylamide) hybrid gels. J Control Release.

[31] Kortesuo P, Ahola M, Kangas M, Leino T, Laakso S, Vuorilehto L (2001). Alkyl-substituted silica gel as a carrier in the controlled release of dexmedetomidine. J Control Release.

[32] Kim HW, Knowles JC, Kim HE (2004). Hydroxyapatite/poly (epsilon-caprolactone) composite coatings on hydroxyapatite porous bone scaffold for drug delivery. Biomaterials.

[33] El-Ghannam A, Ahmed K, Omran M (2005). Nanoporous delivery system to treat osteomyelitis and regenerate bone: Gentamicin release kinetics and bactericidal effect. J Biomed Mater Res B: Appl Biomater.

[34] Volodkin DV, Larionova NI, Sukhorukov GB (2004). Protein encapsulation via porous CaCO_3_ microparticles templating. Biomacromolecules.

[35] Moes AJ (1993). Gastroretentive dosage forms. Crit Rev Ther Drug Carrier Syst.

[36] Despande AA, Rhodes CT, Shah NH, Malick AW (1996). Controlled release drug delivery systems for prolonged gastric residence: An overview. Drug Develop Ind Pharm.

[37] Rouge N, Buri P, Doelker E (1996). Drug absorption sites in the gastrointestinal tract and dosage forms for site specific drug delivery. Int J Pharm.

[38] Hwang SJ, Park H, Park K (1998). Gastric retentive drug delivery systems. Crit Rev Ther Drug Carrier Syst.

[39] Singh BN, Kim KH (2000). Floating drug delivery systems: An approach to oral controlled drug delivery via gastric retention. J Control Release.

[40] Sepulveda P, Binner JG (2005). Processing of cellular ceramics by foaming and in situ polymerization of organic monomers. J Eur Ceram Soc.

[41] Choi SW, Kim JH (2007). Design of surface-modified poly (D,L-lactide-co-glycolide) nanoparticles for targeted drug delivery to bone. J Control Release.

[42] Shen S, Chow PC, Chen F, Tan RB (2007). Submicron particles of SBA-15 modified with MgO as carriers for controlled drug delivery. Chem Pharm Bull.

[43] Ito Y, Kusawake T, Prasad YV, Sugioka N, Sbhita N, Takada K (2006). Preparation and evalution of oral solid gentamycin using emulsifier and adsorbent for in-vitro and in-vivo studies. Int J Pharm.

[44] Patil P, Paradkar A (2006). Porous polystyrene beads as carriers for self-emulsifying system containing loratadine. AAPS Pharm Sci Tech.

[45] Fan YF, Wang YN, Fan YG, Ma FJB (2006). Preparation of insulin nanoparticles and their encapsulation with biodegradable polyelectrolytes via the layer-by-layer adsorption. Int J Pharm.

[46] Xia W, Chang J (2006). Well-ordered mesoporous bioactive glasses (MBG): A promising bioactive drug delivery system. J Control Release.

[47] Jette KK, Law D, Schmitt EA, Kwon GS (2004). Preparation and drug loading of poly (Ethylene Glycol)-block-Poly(ɛ-Caprolactone) micelles through the evaporation of a cosolvent Azeotrope. Pharm Res.

[48] Patil S, Pancholi SS, Agrawal S, Agrawal GP (2004). Surface modified mesoporous ceramics as delivery vehicle for haemoglobin. Drug Deliv.

[49] Gupta MK, Tseng YC, Goldman D, Bogner RH (2002). Hydrogen bonding with adsorbent during storage governs drug dissolution from solid-dispersion granules. Pharm Res.

[50] Queiroz AC, Santos JD, Monteiro FJ, Gibson IR, Knowles JC (2001). Adsorption and release studies of sodium ampicillin from hydroxyapatite and glass-reinforced hydroxyapatite composites. Biomaterials.

[51] Tsung MJ, Burgess DJ (2001). Preparation and characterization of gelatin surface modified PLGA microspheres. AAPS Pharm Sci Tech.

[52] Boer GJ, Kruisbrink J (1987). A polymeric controlled drug delivery device for peptides based on a surface desorption/diffusion mechanism. Biomaterials.

